# Dynamic reciprocal relationships between cognitive and functional declines along the Alzheimer’s disease continuum in the prospective COGICARE study

**DOI:** 10.1186/s13195-021-00887-4

**Published:** 2021-09-03

**Authors:** Sophie Carles, Bachirou O. Taddé, Claudine Berr, Catherine Helmer, Hélène Jacqmin-Gadda, Isabelle Carrière, Cécile Proust-Lima

**Affiliations:** 1 Institute for Neurosciences of Montpellier INM, Univ. Montpellier, INSERM, F-34091 Montpellier, France; 2 Univ. Bordeaux, INSERM, BPH, U1219, F-33000 Bordeaux, France

**Keywords:** Activities of daily living, Cognitive aging, Mini-Mental State Examination, Temporal association, Causality, Dementia

## Abstract

**Background:**

Thoroughly understanding the temporal associations between cognitive and functional dimensions along the dementia process is fundamental to define preventive measures likely to delay the disease’s onset. This work aimed to finely describe the trajectories of cognitive and functional declines, and assess their dynamic bidirectional relationships among subjects at different stages of the dementia process.

**Methods:**

We leveraged extensive repeated data of cognition and functional dependency from the French prospective COGICARE study, designed to better characterize the natural history of cognitive and functional declines around dementia diagnosis. Cognition was measured by the Mini-Mental State Examination, the Isaacs Set Test for verbal fluency, the Benton Visual Retention Test for visuo-spatial memory, and Trail Making Test Part B for executive functioning. Functional dependency was measured by basic and instrumental activities of daily living. The study included 102 cognitively normal, 123 mildly cognitively impaired, and 72 dementia cases with a median of 5 repeated visits over up to 57 months. We used a dynamic causal model which addresses the two essential issues in temporal associations assessment: focusing on intra-individual change and accounting for time.

**Results:**

Better cognitive abilities were associated with lower subsequent decline of the functional level among the three clinical stages with an intensification over time but no reciprocity of the association whatever the clinical status.

**Conclusion:**

This work confirms that the progressive functional dependency could be induced by cognitive impairment. Subjects identified as early as possible with clinically significant cognitive impairments could benefit from preventive measures before the deterioration of activities of daily living and the appearance of dementia clinical signs.

**Supplementary Information:**

The online version contains supplementary material available at 10.1186/s13195-021-00887-4.

## Background

There is now evidence that the pathophysiological process of Alzheimer’s disease (AD) begins decades before the appearance of clinical signs of dementia [1–4]. AD’s preclinical stages may constitute a critical window to define and implement early preventive measures likely to delay the disease’s onset or magnitudes of symptoms. Thoroughly describing the multiple dimensions, mainly cognitive and functional ones, all along the complex AD process, is consequently fundamental. However, longitudinal studies in this field were mainly limited to one dimension at a time ignoring the possible inter-relationships with others. Yet, investigating how the different dimensions interplay over time at distinct clinical stages could be a key to better understand the AD pathological cascade and prioritize interventions.

Uncertainties remain in particular on the temporal relationships between cognitive and functional declines. Subjects with initial clinical presentation of AD have been described with cognitive deficits and no evident limitations in activities of daily living (ADL) that seem to appear later in the disease process [5]. Correlation between cognition and function has been shown to increase as AD patients progressed from preclinical to moderate dementia [6]. Few studies formally assessed the temporal relationships between patterns of cognitive and functional declines and potential differences in their joint evolution along the AD continuum [7–9]. These results suggest that cognitive decline precedes and predicts subsequent functional impairment assessed notably, in mild cognitive impairment (MCI), by the ability to perform ADL. Functional decline has also been suggested as a predictor of both cognitive decline (but only intermittently and not in incident AD cases) [10] and conversion from MCI to AD [11].

Since longitudinal processes are likely to vary between individuals and to change over time, two major aspects have to be taken into account when investigating temporal relationships [12]. First, time is central and association assessment should be unrelated to the visit schedule. Second, the interest should be in the intra-individual change rather than the inter-individual differences. Indeed, from a causal perspective, the goal is to understand how the system changes as time goes on, and what factors influence the future individual change [13]. By relying on autoregressive and cross-lagged models (ARCL), the rare studies investigating reciprocal temporal dependencies between cognition and functional dimensions over time [7, 9, 10] did not account for these two major elements [12]. They considered inter-individual differences between successive levels of variables at observed visit times, thus not targeting intra-individual changes, limiting the interpretation to the visit schedule of the study, and possibly inducing spurious associations if sparse [14]. With cognitive and functional dimensions being measured by scores with ceiling/floor effects or unequal interval scaling [15], a third aspect to take into account is the departure from normality which can induce biased estimates if not properly taken into account [16].

In the current study, we aimed to finely describe the trajectories of both cognitive and functional declines, and assess their dynamic bidirectional relationships among subjects with normal aging, MCI or AD participating in a French cohort study, the natural history of COGnitive decline, and the need of CARE in the elderly (COGICARE) study. We applied for that a dynamic multivariate causal model [17] that overcomes the major methodological requisites to assess temporal associations.

## Methods

### The COGICARE study

The current study analyses the data from COGICARE which is a sub-study of the Three-City (3C) study in Montpellier and Bordeaux centers. COGICARE was designed to better characterize the natural history of cognitive and functional declines around dementia diagnosis through an extensive follow-up of subjects at three different stages: AD, MCI, or cognitively normal (CN). The study protocols of the 3C and COGICARE studies were approved by the Ethics Committee of the University Hospital of Bicêtre and Sud-Méditerranée III (France) and written informed consent was obtained for each participant.

The 3C study is a community-living cohort of 9294 elders (≥ 65 years of age) randomly recruited from the electoral rolls of three French cities (Bordeaux, Dijon, and Montpellier) between 1999 and 2001 [18]. Standardized examinations including face-to-face interviews and cognitive and functional assessments (in a medical center or at home) took place at inclusion and then every 2–3 years (i.e., after 2, 4, 7, 10, 12, and 14 years of follow-up). AD incident cases diagnosis was based on an examination by a neurologist of all participants in Montpellier and of participants suspected of having dementia (based mainly on their clinical, cognitive, or functional assessments) in Bordeaux. An adjudication panel of independent neurologists reviewed all the existing information to confirm the diagnosis of dementia according to the Diagnostic and Statistical Manual of Mental Disorders, 4th edition (DSM-IV) [19], and its etiology. AD was classified according to the National Institute of Neurological and Communicative Disorders and Stroke and the Alzheimer’s Disease and Related Disorders Association (NINCDS/ADRDA) criteria [20]. MCI was defined as (details in Web appendix 1) (i) an alteration of verbal episodic memory (i.e., a free recall score < 17 and a total recall score < 40) [21] on the Free and Cued Selective Reminding Test (FCSRT) [22] and (ii) partial or total limitation in their abilities to perform at least two of four Instrumental Activities of Daily Living (IADL) [23].

The following were eligible to enter the COGICARE sub-study: (1) AD incident cases from Bordeaux and Montpellier centers diagnosed at the 3C 7- or 9-year follow-up and non-demented at the last visit preceding the AD diagnosis. Each incident AD case was theoretically matched to one control considered as cognitively normal (CN) from the pool of participants free of both dementia and MCI according to sex, age (+/−2.5 years), and examination date (within the interval of 45 days before or after the diagnosis date of their matched AD case); (2) participants who fulfilled criteria for MCI at the 3C 9-year follow-up.

Of the 4363 participants included in the Bordeaux’s and Montpellier’s centers, 2751 and 2409 were still followed at the 3C 7- and 9-year follow-up, respectively. Among all incident AD cases at the 7- (*n* = 133) and 9-year (*n* = 114) follow-up, 176 were included in COGICARE (participation rate: 71%). Furthermore, 166 MCI identified at the 3C 9-year follow-up were included. A total of 125 matched CN controls were included. The 467 COGICARE participants (AD, MCI, or CN), underwent cognitive and functional assessments every 6 months from the 3C 9-year follow-up (thereafter considered as baseline) and during 18–24 months and then underwent their planned 3C follow-ups.

### Cognitive and functional assessments

Three cognitive tests were intensively administered during COGICARE follow-up. The global cognitive function was assessed using the face-to-face 30-item Mini-Mental-State-Examination (MMSE) [24] for AD and MCI, and the telephonic 25-item MMSE for CN. Telephonic 25-item MMSE scores were rescaled so that all MMSE scores ranged from 0 (severe impairment) to 30 (no impairment). The verbal fluency was assessed by the Isaacs Set Test (IST) truncated at 30 s [25] which was either administered face-to-face for AD and MCI or by telephone for the CN. Its range was 0 to 82. The visuo-spatial memory was assessed by the Benton Visual Retention Test (BVRT) [26] (range 0–15) during face-to-face interviews for AD and MCI. In addition, we considered the Trail Making Test Part B (TMT-B) [27] administered only during the 3C visits (twice during this study period) in order to include a measure of executive functioning to the cognitive level definition. The score was the number of correct moves per minute.

Functional dependency was assessed by the limitation in basic ADL (BADL) scale [28] and Instrumental ADL (IADL) scale [23] during face-to-face (for AD and MCI) and telephonic interviews (for CN). A total of 5 BADL (bathing, dressing, toileting, eating, and transferring) and 4 IADL (telephone use, transportation, self-administration of medication, finances) were considered [29], each one on a 3-point scale as having no limitation, partial limitation, or total limitation (coded 0, 1, and 2 respectively) in performing the task. Following a previous work that showed a continuum in IADL and BADL [30], we considered the total sumscore of IADL and BADL which ranged from 0 to18.

### Study sample within COGICARE

From the 467 participants of the COGICARE study, we excluded the individuals with no repeated cognitive or functional measures during the 57 months of follow-up considered for the present study, and with incomplete information on potential confounders (Flowchart in Web Figure 1). Potential confounders were age at baseline (3C 9-year follow-up) in 5 categories chosen at the quintiles of the distribution (≤ 79.1 as the reference class, 79.1–82.0, 82.0–84.5, 84.5–87.7, ≥ 87.7), sex, binary educational level (lower/equal to primary school versus higher than primary school), and Apolipoprotein E (apoE) ε4 status (at least one ε4 allele versus none). The final study sample included 297 individuals.

### Statistical analysis

**Fig. 1:**
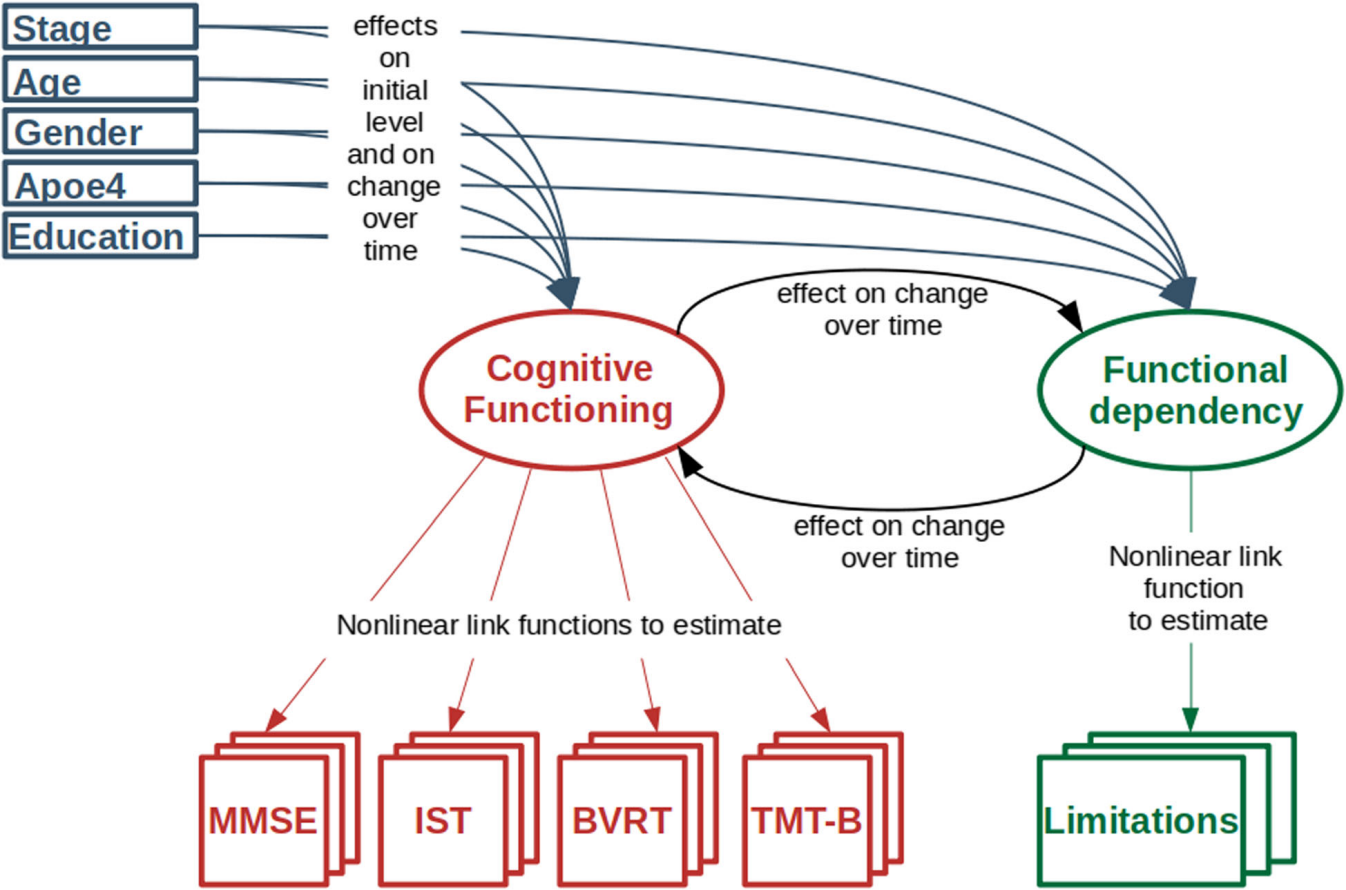
Theoretical graph of the dynamic causal model

Trajectories of cognitive and functional declines and their temporal reciprocal relationships according to the clinical status (AD, MCI, CN) were assessed using a multivariate latent process model [17]. The theoretical graph of the model is specified in Fig. 1.

The model describes the trajectories and inter-dependencies over time of the cognitive functioning and functional dependency, defined as two latent processes. Cognitive process was measured by MMSE, IST, BVRT, and TMT-B while functional process was measured by the BADL-IADL sum score. In order to correct for its departure from normality and ceiling/floor effect, each observed marker was linked to its underlying latent process using a marker-specific parameterized link function approximated by quadratic I-splines with two internal knots chosen at the quartiles for the MMSE, IST, BVRT, and TMT-B, and one internal knot at the median for the limitation score [31]. The latent processes were standardized (mean 0, standard deviation 1) at baseline in the reference category (CN men with a higher educational level and less than 79.1 years old at baseline). Thus, in the results section, a one-unit change of cognition or functional dependency always corresponds to the residual standard deviation of the dimension at baseline after adjustment for age, sex, age, and clinical stage.

The longitudinal model for the cognitive and functional latent trajectories was split in two subparts to exhibit the temporal relationships. Both parts accounted for intra-individual correlation and missing at random mechanism (as based on the mixed model theory [32]):

- The initial levels of cognitive ability and functional dependency were regressed on the stage of the disease, age, apoE4 status, gender, education, the interaction between education and stage, and an individual random intercept.
- The changes over time of cognitive ability and functional dependency were described over a finely discretized time of 3 months unrelated to the visit process. The change between *t* and *t* + 3 months was regressed on the same covariates as initial levels, an individual random effect, and the level of cognitive ability and functional dependency at time t. The effect of the level of cognitive ability and functional dependency on each other was different by stage and could vary over time to precisely explore their inter-relations, and especially potential changes in these relationships over time. Further details on the dynamic model specification are given in Web Appendix 2.

Statistical analyses were done with R software and the dynamic model was fitted using CInLPN R package available at https://github.com/bachirtadde/CInLPN. Reported *P* values are from two-sided Wald tests.

## Results

### Sample description

The study sample included 102 CN (34.3%), 123 were MCI (41.4%), and 72 were AD (24.3%). AD subjects (median age 84.4 years) were slightly older than MCI (82.0 years) and CN (83.4 years) at inclusion (Table 1). Two third of the sample were women with slight differences over groups (65.3%, 58.5%, and 67.6% for AD, MCI, and CN, respectively). MCI and AD subjects had a lower educational level compared to CN. Subjects with AD were more frequently APOE ε4 carriers than CN (36.1% vs 14.7%). The percentage of individuals with no IADL and BADL limitations at baseline gradually decreased between CN, MCI, and AD, and MCI and AD had lower cognitive scores than CN.

**Table 1:** Characteristics of participants at baseline (*n* = 297), COGICARE study

	CN	MCI	AD	All
	(*N* = 102)	(*N* = 123)	(*N* = 72)	(*N* = 297)
Age in years: median (min–max)	83.4 (75.6–92.8)	82.0 (74.2–95.2)	84.4 (76.0–93.5)	82.9 (74.2–95.2)
Male sex (%)	32.35	41.46	34.72	36.70
Education level higher than primary school (%)	77.45	65.85	62.50	69.02
ApoE4 carriers (%)	14.71	13.82	36.11	19.53
Limitation in activities of daily living (%):				
Bathing				
No limitation	96.08	95.93	66.67	88.89
Partial limitation	1.96	2.44	16.67	5.72
Total limitation	1.96	1.63	16.67	5.39
Dressing				
No limitation	96.08	97.56	73.61	91.25
Partial limitation	0.98	1.63	13.89	4.38
Total Limitation	2.94	0.81	12.50	4.38
Toileting				
No limitation	100.00	99.19	95.83	98.65
Partial limitation	0.00	0.00	4.17	1.01
Total limitation	0.00	0.81	0.00	0.34
Transfering				
No limitation	99.02	99.19	94.44	97.98
Partial limitation	0.98	0.81	5.56	2.02
Total limitation	0.00	0.00	0.00	0.00
Eating				
No limitation	99.02	100.00	95.83	98.65
Partial limitation	0.98	0.00	4.17	1.35
Total limitation	0.00	0.00	0.00	0.00
Limitation in instrumental activities of daily living (%):				
Telephone use				
No limitation	100.00	95.12	56.94	87.54
Partial limitation	0.00	4.88	36.11	10.77
Total limitation	0.00	0.00	6.94	1.68
Shopping				
No limitation	78.43	77.24	36.11	67.68
Partial limitation	13.73	19.51	26.39	19.19
Total limitation	7.84	3.25	37.50	13.13
Transportation				
No limitation	79.41	83.74	44.44	72.73
Partial limitation	19.61	15.45	48.61	24.92
Total limitation	0.98	0.81	6.94	2.36
Medication				
No limitation	99.02	95.12	48.61	85.19
Partial limitation	0.00	4.07	25.00	7.74
Total limitation	0.98	0.81	26.39	7.07
Finances				
No limitation	89.22	86.99	41.67	76.77
Partial limitation	10.78	11.38	26.39	14.81
Total limitation	0.00	1.63	31.94	8.42
Cognitive scores: median (min–max)				
MMSE	28 (17–30)	27 (20–30)	23 (11–28)	27 (11–30)
IST	45 (22–82)	39 20–61)	29 (12–62)	39 (12–82)
BVRT	12 (6–15)	11 (5–15)	9 (5–14)	11 (5–15)
TMT-B	26.5 (3.0–60.0)	21.3 (0.5–62.6)	7.3 (0.7–40.0)	20.0 (0.5–62.6)
Limitation score: median (min–max)	0 (0–7)	0 (0–14)	3 (0–13)	0 (0–14)
Number of measures by subject over follow-up: median (min–max)				
MMSE	5 (2–6)	5 (2–7)	4 (1–7)	5 (1–7)
IST	4 (1–6)	5 (1–7)	4 (1–7)	4 (1–7)
BVRT	2 (1–4)	5 (2–6)	3 (1–7)	3 (1–7)
TMT-B	2 (1–2)	1 (1–2)	1 (1–2)	1 (1–2)
Limitations	5 (2–6)	5 (2–7)	5 (2–7)	5 (2–7)

The 170 excluded subjects (see flowchart in Web Figure 1) were more likely AD (61.2% AD, 13.5% MCI, 25.3% CN). They were slightly older (median age: 86.5) and more likely to be women (72.9%) than included subjects but comparable regarding their education level and APOE ε4 status.

The sample comprised a total of 3974 and 1388 repeated measures of cognition (through the 4 scores) and function, respectively. The median number of visits per participant was 5 for all groups. Web Table 1 and Web Figure 2 further describe the number of measurements available by clinical stage and 3-month steps and the observed individual marker trajectories.

### Cognitive and functional trajectories

**Fig. 2:**
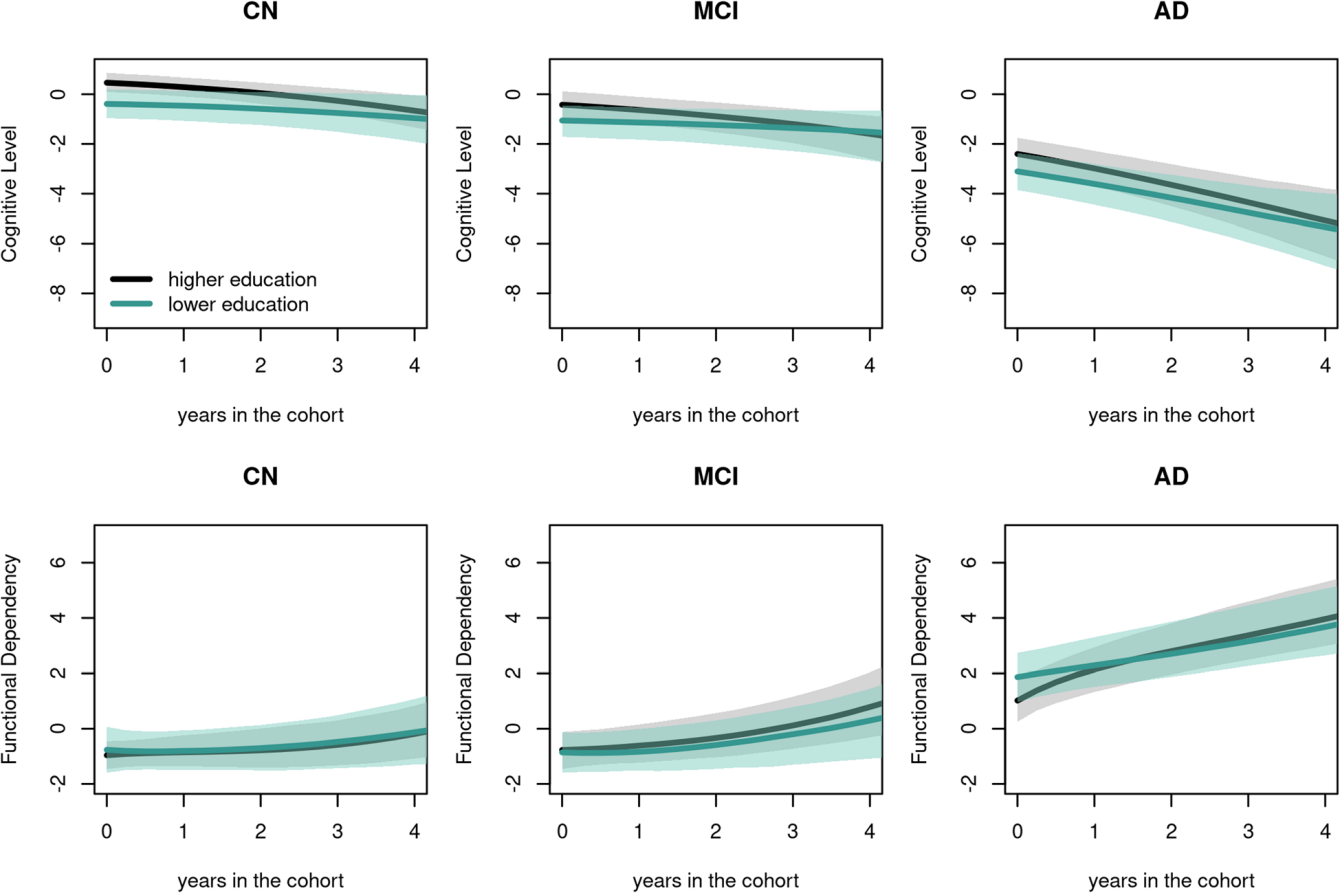
Predicted mean trajectories over time (plain line) and associated 95% confidence intervals (shades) of the underlying cognitive ability and functional dependency according to the clinical stage (CN, MCI, and AD) and education; trajectories are displayed for non-APOE ε4 carriers; male sex and aged between 82.60 and 85.12. Ninety-five percent confidence intervals were obtained using a Monte Carlo method with 2000 draws

The mean predicted trajectories of cognition and function showed a gradual progression from normal to AD stage with differences according to education (Fig. 2). Estimations from the dynamic model for cognitive and functional trajectories are reported in Table 2 for the baseline levels and in Table 3 for the rates of change and further described below. The association between observed markers and the underlying dimensions highlighted the curvilinear nature of the markers and notably confirmed the ceiling effect of MMSE and the floor effect of TMT-B (See Web Figure 3). The adequacy of the model to the data was very good (see Web Figure 4 for the comparison of predictions with observations).

**Table 2:** Estimated associations with the underlying cognitive ability and functional dependency level at baseline (*n* = 297), COGICARE study

	Cognitive ability at baseline			Functional dependency at baseline		
	Coefficient*	95% confidence interval		Coefficient*	95% confidence interval	
Clinical stage by education level						
Higher than primary school						
CN	0	-		0	-	
MCI	− 0.882	− 1.284	− 0.480	0.171	− 0.275	0.617
AD	− 2.878	− 3.419	− 2.337	1.974	1.415	2.532
Lower or equal to primary school						
CN	− 0.868	− 1.308	− 0.429	0.188	− 0.417	0.793
MCI	− 1.531	− 2.044	− 1.018	0.089	− 0.412	0.591
AD	− 3.572	− 4.219	− 2.926	2.811	2.105	3.518
Age at baseline						
≤ 79.1	0	-		0	-	
79.1–82.0	− 0.284	− 0.680	0.113	0.069	− 0.396	0.534
82.0–84.5	− 0.461	− 0.870	− 0.053	0.304	− 0.167	0.775
84.5–87.7	− 0.288	− 0.691	0.116	0.284	− 0.200	0.767
> 87.7	− 0.754	− 1.146	− 0.362	1.028	0.508	1.549
APOE ε4 carrier						
No						
Yes	− 0.010	− 0.351	0.331	− 0.355	− 0.741	0.031
Sex						
Male						
Female	− 0.016	− 0.270	0.238	0.316	− 0.017	0.650

*One-unit difference corresponds to the standard deviation of the dimension at baseline in the category reference (CN men with a higher educational level and less than 79.6 years old at baseline)

**Table 3:** Estimated associations with the rate of change of underlying cognitive ability and functional dependency (*n* = 297), COGICARE study

	Change over time of cognitive ability			Change over time of functional dependency		
	Coefficient*	95% CI		Coefficient*	95% CI	
Intercept	− 0.247	− 0.371	− 0.122	0.201	− 0.370	0.772
Clinical stage						
CN	0	-		0	-	
MCI	0.176	0.021	0.332	0.194	− 0.223	0.611
AD	0.377	− 0.031	0.785	2.166	0.784	3.549
Age at baseline						
≤ 79.6	0	-		0	-	
79.60–82.60	− 0.021	− 0.165	0.124	− 0.144	− 0.552	0.264
82.60–85.12	− 0.040	− 0.191	0.110	− 0.091	− 0.508	0.325
85.12–88.34	0.003	− 0.141	0.148	0.175	− 0.248	0.599
> 88.34	0.043	− 0.119	0.204	0.732	0.114	1.349
APOE ε4 carrier						
No	0	-		0	-	
Yes	− 0.017	− 0.139	0.104	− 0.211	− 0.582	0.159
Sex						
Male	0	-		0	-	
Female	0.061	− 0.036	0.157	0.117	− 0.201	0.436
Education level						
> Primary school	0	-		0	-	
≤ Primary school	0.239	0.124	0.355	− 0.471	− 0.828	− 0.114
Current functional level**						
CN	− 0.086	− 0.254	0.082	− 1.105	− 1.943	− 0.267
MCI	− 0.010	− 0.141	0.122	− 0.643	− 1.247	− 0.038
AD	− 0.032	− 0.160	0.096	− 0.877	− 1.484	− 0.269
CN × time	0.032	− 0.035	0.100			
MCI × time	− 0.008	− 0.057	0.042			
AD × time	0.033	− 0.003	0.069			
Current cognitive level**						
CN	0.154	0.052	0.255	− 0.403	− 0.783	− 0.023
MCI	0.185	0.081	0.290	− 0.307	− 0.609	− 0.006
AD	0.235	0.130	0.340	− − 0.342	− 0.601	− − 0.083
CN × time				− 0.281	− 0.468	− 0.095
MCI × time				− 0.215	− 0.413	− 0.018
AD × time				− 0.043	− 0.099	0.014

*One unit corresponds to an annual change of the same size as one standard deviation of the dimension at baseline in the reference category (CN men with a higher educational level and less than 79.6 years old at baseline)

**One unit corresponds to the standard deviation of the dimension at baseline in the reference category (CN men with a higher educational level and less than 79.6 years old at baseline)

#### Association with baseline cognitive and functional levels

A gradient in baseline cognitive and functional abilities was observed according to the clinical stage after adjustment for other potential confounding factors, with slight differences according to education. Participants with an educational level higher than primary school had systematically a higher cognitive level in mean compared to low educated participants with no substantial differences across groups (mean difference in the latent dimension scale (MD) = − 0.868, 95% confidence interval (95%CI) =− 1.308, − 0.429, *P* = 0.0001 for CN; MD = − 0.649, 95%CI = − 1.013, − 0.285, *P* = 0.0005 for MCI; and MD = − 0.695, 95%CI = − 1.178, − 0.211, *P* = 0.005 for AD). For functional dependency, differences according to education were only observed among AD subjects, lower educated AD subjects having higher limitations than higher educated AD subjects (MD = 0.838, 95%CI = 0.237, 1.439, *P* = 0.006). Adjusted for clinical stage and other potential confounding factors, there were no differences in cognition according to APOE ε4 status (*p* = 0.954) and gender (*p* = 0.903). However, APOE ε4 carriers tended to have a better functional ability (MD = − 0.355, 95%CI = − 0.741,0.0310, *P* = 0.072) at baseline compared to non-carriers, and so did men (MD = − 0.316, 95%CI = − 0.650, 0.017, *P* = 0.063). Older individuals were more cognitively and functionally impaired adjusted for the other factors.

#### Association with cognitive and functional rates of change

**Fig. 3:**
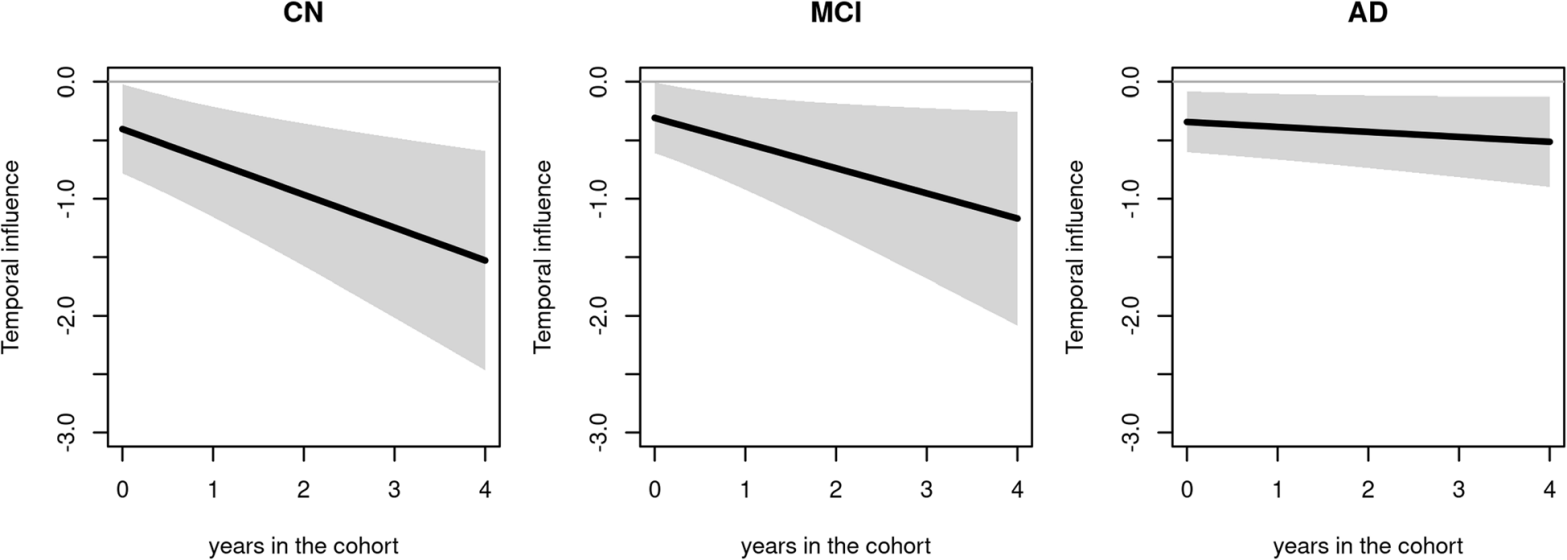
Estimated trajectory of temporal influence (and associated 95%CI) of the cognitive level on the subsequent functional dependency change according to the time in the cohort by clinical status (CN, MCI, and AD). The reported temporal influence corresponds to the difference in the annual change of functional dependency for a one-unit increase of the current cognitive level

Adjusted for confounding factors, the functional level at any time t was not significantly associated with the subsequent rate of change in cognitive dimension in any group (all p> 0.320). In contrast, whatever the clinical stage, the cognitive level significantly affected the subsequent rate of change of functional dependency after adjustment on potential confounding factors (in all groups: p< 0.045 at baseline, p< 0.009 at 2 years, p< 0.012 at 4 years). As illustrated in Fig. 3, higher cognitive abilities at any given time were systematically associated with a lower subsequent change in functional limitations with an intensification of the association over time for CN subjects and to a lesser extent for MCI (and even lesser extent for AD). This means that the benefit of better cognitive abilities on the change of limitation was larger and larger as the time spent in the cohort increased. The annual change in functional limitations was reduced by 0.403 (95%CI = 0.023, 0.783, *P* = 0.037), 0.966 (95%CI = 0.355, 1.576, *P* = 0.002), and 1.529 (95%CI = 0.591, 2.466, *P* = 0.001) for a one-unit increase of current cognition in CN subjects after 0, 2, and 4 years in the cohort, respectively. It was reduced by 0.307 (95%CI = 0.006, 0.609, *P* = 0.045), 0.738 (95%CI = 0.184, 1.293, *P* = 0.009), and 1.169 (95%CI = 0.255, 2.084, *P* = 0.012) for a one-unit increase of current cognition in MCI subjects after 0, 2, and 4 years, respectively. It was reduced by 0.342 (95%CI = 0.083, 0.601, *P* = 0.010), 0.427 (95%CI = 0.118,0.736, *P* = 0.007), and 0.512 (95%CI = 0.126, 0.899, *P* = 0.009) for a one-unit increase of current cognition in AD subjects after 0, 2, and 4 years in the cohort, respectively.

The size of these temporal associations can be compared with others. For instance, for the same current cognitive level, and the same other confounding factors, the annual change of functional limitations was reduced by 1.105 (95%CI = 0.267, 1.943, *p* = 0.010), 0.643 (95%CI = 0.038, 1.247, *p* = 0.037), and 0.877 (95%CI = 0.269, 1.484, *p* = 0.005) for a one-unit increase of current functional limitation in CN, MCI, and AD, respectively. The annual increase in functional limitations was also less pronounced for low educated subjects compared to higher educated subjects with a constant mean difference of − 0.471 (95%CI = − 0.828, − 0.114, *P* = 0.010).

Adjusted for current cognitive and functional levels and other confounding factors, there was no residual association with an annual change of cognition or limitation for gender, APOE ε4, and age except for the oldest (> 88.3 years old) who had a higher increase of functional dependency than younger participants. There was also a residual association of clinical status with functional change, AD individuals having a much higher functional annual change (MD = 2.166, 95%CI = 0.784, 3.549, *P* < 0.002) than CN and MCI.

## Discussion

Leveraging longitudinal cohort data and exploiting a dynamic statistical model dedicated to the evaluation of temporal associations, this work allows understanding the cognitive and functional trajectories over time along the AD continuum, from normal cognitive aging to MCI and AD, and assesses the temporal relationships between cognitive and functional dimensions. Our study showed that better cognitive abilities were associated with a lower subsequent decline of the functional level among CN, MCI, and AD, but with no reciprocity whatever the clinical stage. We adjusted on main confounders identified in cognitive aging studies but cannot exclude residual confounding in these relationships.

Our results are consistent with part of the few studies that explored the potential reciprocal causal-effect between cognitive decline and functional impairment and showed that in MCI subjects or non-demented community-dwelling older persons the cognitive impairment preceded and predicted subsequent functional decline but functional impairment did not predict cognitive decline [7–9, 33, 34]. Although Zahodne et al. [10] also concluded to a main causal effect of the cognitive impairment on the functional decline, they reported more mixed findings with a causal effect of the functional decline on the cognitive impairment also observed at a few times in non-demented elders and prevalent AD but not in incident AD. Several other studies have not formally explored the dynamic bi-directional association, but rather described the trajectories of cognitive and functional abilities and compared the shape of the declines in the pre-dementia phase. They identified that the decline in cognitive performances (observed first in measures of semantic memory and conceptual formation, and then in more global cognitive functioning) preceded the increase in dependency in IADL and in BADL [1, 35].

From a methodological point of view, differences in the literature results could first be attributable to heterogeneity in the scales used to assess cognitive and functional dimensions, some functional scales including for instance a social dimension. Second, previous works used ARCL models which focus on (i) determinants of inter-individual differences over time rather than determinants of intra-individual changes [12, 13] and (ii) differences between observed visits in the study rather than differences in continuous [12–14] or finely discretized [17] time which are yet necessary for causal interpretations. The use of ARCL models makes previous results strongly dependent on the visit schedule, possibly too sparse for causal interpretation, and different from one study to the other so that comparisons are challenging. It also assumes that processes do not substantially change over time at the individual level which is very unlikely. In our work, we circumvented these limits by exploring associations with the subsequent individual rate of change of each dimension and retrieving association over a 3-month period which is unrelated to the visit schedule and very small compared to the AD process timescale. By relying on the mixed model theory, the method naturally handled intermittent and monotonic missing data under the missing at random mechanism [36]. We also considered that the temporal relationships may evolve between CN, MCI, and AD, and over time within each group, thus allowing the identification of specific time windows where the cognitive/functional dimensions could have a more important influence on the evolution of the other. We underlined that the cognitive latent dimension influenced the functional change at each stage of the disease and at each time window of interest. However, the influence of high cognitive abilities in maintaining the functional abilities increased over time, particularly in cognitively normal and MCI subjects.

A major strength of this work is the repeated longitudinal data collected in the COGICARE study. Exploring subtle changes in the dynamic of cognitive and functional dimensions was made possible by the regular follow-up of elders along the disease process. In this study, the MCI and AD groups were precisely defined. Subjects with AD were identified through an active screening and confirmed by an independent experts committee. The potential of a misclassification bias is thus minimized. The threshold used to identify MCI has also been shown to discriminate MCI who will develop AD from MCI non-converters [21]. Targeting these subjects offered the opportunity to better characterize the prodromal period of AD. When exploring temporal relationships, the number of repeated measures is also a critical issue. The median number of visits per subject in our study was 5 in all groups both for the cognitive and functional dimensions. It is particularly valuable for AD as the disease is often closely associated with attrition that can potentially introduce bias in the results and lower the possibility to assess the dynamic of processes. Finally, the cognitive dimension was defined from an extensive battery of cognitive tests including global functioning, visuo-spatial memory, verbal fluency, and executive functioning, domains particularly central in cerebral aging. The definition of a general latent cognitive factor using these specific domains had been previously validated by the authors [37].

## Limitations

This study has some limitations. First, some psychometric tests (BVRT for CN and TMT-B for all groups) were only assessed during the 3C interviews. Considering TMT-B substantially reduced the size of the sample (112 participants did not have any TMT-B evaluation). However, it was critical that the cognitive dimension included an assessment of executive functioning given the robust relationship between instrumental activities of daily living and tests of executive function repeatedly identified in the literature (e.g., [38]).

Second, at COGICARE visits, MMSE and IST scores resulted from telephonic interviews among controls and from face-to-face interviews in MCI and AD subjects. However, it has been shown that telephonic version of MMSE provides reliable results [39]. In addition, the shape of the latent cognitive process trajectories over time among controls seems coherent with previous studies [1, 40]. Functional dependency was assessed through a summary score of 4 instrumental activities of daily living and 5 basic activities of daily living following previous works that showed a continuum of degradation in these items [29, 30]. However, by focusing on (instrumental) activities of daily living, we might have missed very early changes in functional dependency. Finally, we considered that data were missing at random and we did not take into account the possible changes of status over time. Yet, by the end of the follow-up, 26 of the 123 MCI had become AD, and 24/8 of the 102 controls had become MCI/AD. Therefore results must be interpreted for groups of subjects initially healthy, MCI or AD.

## Conclusion

The approach we used provides valuable information on the dynamic co-evolution of the cognitive and function dimensions along the AD continuum. This work supports the hypothesis that cognitive performances contribute to maintain functional abilities whether subjects are cognitively normal or in the pathological process of cognitive decline. As stated in a recent FDA report [41], such a result is an essential argument to reinforce persuasiveness of the clinical trials identifying treatment effect on cognitive functioning. We highlighted that the benefit of cognitive performances in maintaining functional abilities increased over time when subjects were cognitively normal or at the beginning of the pathological process (MCI) but not at later stages (AD) when the severity of the disease is probably so high that function cannot be maintained. In the absence of a cure for AD, a pivotal challenge is to maintain a good quality of life for future AD as long as possible without overwhelming functional abilities. Identifying subjects with significant cognitive impairments as early as possible is consequently crucial so that they can benefit from preventive measures before the appearance of AD clinical signs.

## Supplementary Information

**Additional file 1.**

## Data Availability

Anonymized data will be shared by reasonable request to the 3C scientific committee. Scripts for replicating the analyses can be sent on demand.
