# Supplementary material for: Dynamic reciprocal relationships between cognitive and functional declines along the Alzheimer’s disease continuum in the prospective COGICARE study
Source: Alzheimers Res Ther. 2021 Sep 3;13:148. doi: 10.1186/s13195-021-00887-4 (PMC8418020; doi:10.1186/s13195-021-00887-4)

Web Material for Dynamic Reciprocal relationships between cognitive and functional declines along the AD continuum in the prospective COGICARE study

(manuscript submitted to publication in *Alzheimer's Research & Therapy*)

Sophie Carles[1], Bachirou O.Taddé[2] , Claudine Berr[1], Catherine Helmer[2], Hélène Jacqmin-Gadda[2], Isabelle Carrière[1], Cécile Proust-Lima[2]

[1] Institute for Neurosciences of Montpellier INM, Univ. Montpellier, INSERM, F-34091 Montpellier, France

[2] Univ. Bordeaux, INSERM, BPH, U1219, F-33000 Bordeaux, France

*corresponding author: cecile.proust-lima@inserm.fr ; ORCID: 0000-0002-9884-955X

Web Appendix 1: detailed MCI definition

MCI status at entry in COGICARE study was defined according to two criteria:

(i) an alteration of verbal episodic memory (i.e. a free recall score <17 and a total recall score <40) [1] on the Free and Cued Selective Reminding Test (FCSRT) [2]. During FCSRT, after an encoding phase (reading and memorization of 16 words), the participant is immediately asked by a trained neuropsychologist to quote as many words as possible. Then, for the uncited words, a cue is provided (semantic category). The free and cued recalls are repeated three times and provide a free recall score (number of words retrieved at the three free recall trials) and a total recall score (number of words retrieved by free and cued recalls) expressed on a 0 (no recall) to 48 (complete recall) scale.

(ii) partial or total limitation in their abilities to perform at least two of the four Instrumental Activities of Daily Living (IADL) of the Lawton and Brody scale [3].

Web Appendix 2 : Dynamic causal model specification

We detail in this paragraph how the dynamic causal model of Taddé et al. [4] was specified in the analysis of cognitive and functional dimensions. The vector $y_{id}=\left(y_{i1d},\dots,y_{in_{id}d,}\right)^T$ denotes a n_{id} vector of observed measures of marker d for individual i at observation time t_{ijd}, $i=1,\dots,N$, $j=1,\dots,n_{id}$, $d=1,\dots,D$. Specifically, the D=5 markers under study are the MMSE score $(d=1)$, IST score $(d=2)$, BVRT score $(d=3)$ and TMT-B score $(d=4)$ for dimension Cognitive functioning (C) and the limitation score based on IADL and ADL $(d=5)$ for dimension Functional dependency (F). The times and number of times can be different from one subject to the other and from one marker to the other.

Main manuscript Figure 1 provides the theoretical graph of the model. For each individual i, vectors of observed measures $y_{i1}, y_{i2}, y_{i3}, y_{i4}$ are considered as noisy measures of the underlying cognitive process $\left(\Lambda_i^C(t)\right)$ and the vector of observed measures y_{i5} provides noisy measures of the underlying functional process $\left(\Lambda_i^F(t)\right)$. In order to correct for their departure from normality and ceiling effects, each marker is linked to its underlying latent process using a link function H_d so that $H_d(y_{ijd}) = \Lambda_i^C(t_{ijd}) + e_{ijd}$ for $d = 1, \ldots, 4$ and $H_5(y_{ij5}) = \Lambda_i^F(t_{ij5}) + e_{ij5}$ with e_{ijd} Gaussian independent measurement errors for $d = 1, \ldots, 5$. Each link function H_d is approximated by quadratic I-splines with two internal knots chosen at the quartiles of the score distribution for cognitive scores, and one internal knot at the median of the limitation score distribution.

To retrieve temporal associations, time $t = s \times \delta$ is discretized with δ a constant discretization step of 3 months which has been shown to be very small in regards with the progression of the pathology [4] and limits the number of duplicated measures within a same time interval. The correspondence is such that $s = 0$ for baseline measures $(t_{ijd} = 0)$, $s = 1$ for $0 < t_{ijd} \leq 3$, $s = 2$ for $3 < t_{ijd} \leq 6$ and so on.

The structural model for the trajectories of the processes is split in two parts.

(i) The latent processes at baseline $(t = 0)$, $\Lambda_i^C(0)$ and $\Lambda_i^F(0)$ are modeled with a multivariate linear mixed model as follows:

$$\begin{aligned}\Lambda_i^d(0) = {} & \beta_0^d + \beta_1^d MCI_i + \beta_2^d AD_i + \beta_3^d AGE0_{1i} + \beta_4^d AGE0_{2i} + \beta_5^d AGE0_{3i} + \beta_6^d AGE0_{4i} + \beta_7^d Apoe4_i \\ & + \beta_8^d Educ_i + \beta_9^d Gender_i + \beta_{10}^d Educ_i x\, MCI_i + \beta_{11}^d Educ_i x\, AD_i + u_i^d \\ & \text{with } d = (C, F)\end{aligned}$$

Initial levels $\Lambda_i^d(0)$ (for $d = (C, F)$) are related to the vector of explanatory variables including the intercept, clinical stage (AD or MCI; controls being the reference group) and potential confounders : age at baseline $AGE0$ considered in 5 categories (≤79.60 as the reference class,]79.60-82.60],]82.60-85.12],]85.12-88.34], ≥88.34) due to the non linear association

between age and the system of latent processes at baseline; APOE ε4 status; education level (higher than primary school as the reference versus end of primary school at most); gender (male in reference); and interactions between education level and clinical stage. Each dimension-specific individual random intercept u_i^d follows a Gaussian distribtion with variance 1. Random intercepts are correlated across dimensions to account for possible associations between dimensions before baseline.

(ii) the rate of change of each process $\left(\frac{\Delta \Lambda_i^d(t+\delta)}{\delta}=\frac{\Lambda_i^d(t+\delta)-\Lambda_i^d(t)}{\delta}\right)$ between two successive discrete times (t and $t+\delta$) is modelled by difference equation combined with a multivariate linear mixed model:

$$\frac{\Delta \Lambda_i^C(t+\delta)}{\delta}=\gamma_0^C+\gamma_1^C MCI_i+\gamma_2^C AD_i+\gamma_3^C AGE\,0_{1i}+\gamma_4^C AGE\,0_{2i}+\gamma_5^C AGE\,0_{3i}+\gamma_6^C AGE\,0_{4i}+\gamma_7^C Apoe\,4_i$$
$$+\gamma_8^C Educ_i+\gamma_9^C Gender_i+v_i^C+\left(\alpha_{CC}^0+\alpha_{CC}^1 MCI_i+\alpha_{CC}^2 AD_i\right)\Lambda_i^C(t)$$
$$+\left(\alpha_{CF}^0(t)+\alpha_{CF}^1(t)\,MCI_i+\alpha_{CF}^2(t)\,AD_i\right)\Lambda_i^F(t)$$

$$\frac{\Delta \Lambda_i^F(t+\delta)}{\delta}=\gamma_0^F+\gamma_1^F MCI_i+\gamma_2^F AD_i+\gamma_3^F AGE\,0_{1i}+\gamma_4^F AGE\,0_{2i}+\gamma_5^F AGE\,0_{3i}+\gamma_6^F AGE\,0_{4i}+\gamma_7^F Apoe\,4_i$$
$$+\gamma_8^F Educ_i+\gamma_9^F Gender_i+v_i^F+\left(\alpha_{FC}^0(t)+\alpha_{FC}^1(t)\,MCI_i+\alpha_{FC}^2(t)\,AD_i\right)\Lambda_i^C(t)$$
$$+\left(\alpha_{FF}^0+\alpha_{FF}^1 MCI_i+\alpha_{FF}^2 AD_i\right)\Lambda_i^F(t)$$

Rates of change $\frac{\Delta \Lambda_i^d(t+\delta)}{\delta}$ are related to a vector of covariates that includes intercept, clinical stage and the same potential confounders as for initial levels. The individual random

intercepts $v_{1,i}^{d}$ represent the individual departure to the mean rate of change for dimension d. Within-dimension random effects (u_{i}^{d} and $v_{1,i}^{d}$) were correlated to flexibly account for intra-individual correlation over time but random effects were considered independent between dimensions (except for u_{i}^{d} as specified above).

Elements α are components of the matrix of transition intensities that models the reciprocal temporal relationships between processes. For instance $\alpha_{\dot{C}F}(t)$ quantifies the temporal effect of the functional process F at time t on the rate of change of the cognitive process C between t and $t+\delta$ while $\alpha_{\dot{F}C}(t)$ quantifies the temporal effect of the cognitive process C at time t on the rate of change of the functional process F between t and $t+\delta$. According to our main question of interest we considered that the temporal dependencies could fluctuate for AD, MCI and controls by introducing the corresponding covariates in the modeling of the transition matrix. We also considered that the cross effects of the cognitive process and functional process at time t on the rate of change of the other process between two successive discrete times, that is $\alpha_{\dot{F}C}(t)$ and $\alpha_{\dot{F}C}(t)$, respectively, were different linear functions of time in each group (CN, MCI and AD).

The parameters of the model were obtained by Maximum Likelihood using function CinLPN function of CinLPN R package available at : https://github.com/bachirtadde/CInLPN.

References

1. Sarazin M, Berr C, De Rotrou J, Fabrigoule C, Pasquier F, Legrain S, et al. Amnestic syndrome of the medial temporal type identifies prodromal AD: a longitudinal study. Neurology. 2007;69:1859–67.

2. Grober E, Sanders AE, Hall C, Lipton RB. Free and cued selective reminding identifies very mild dementia in primary care. Alzheimer Dis Assoc Disord. 2010;24:284–90.

3. Lawton MP, Brody EM. Assessment of older people: self-maintaining and instrumental activities of daily living. Gerontologist. 1969;9:179–86.

4. Taddé BO, Jacqmin-Gadda H, Dartigues J-F, Commenges D, Proust-Lima C. Dynamic modeling of multivariate dimensions and their temporal relationships using latent processes: Application to Alzheimer's disease. Biometrics. 2020;76:886–99.

Web Table 1: Description of available cognitive and functional measures by clinical stage group from the Cogicare study (n=297), France

No. of measures	MMSE			IST			BVRT			TMT-B			Limitation score		
	CN	MCI	AD	CN	MCI	AD	CN	MCI	AD	CN	MCI	AD	CN	MCI	AD
Whole Study Period	494	564	295	354	553	288	229	548	239	157	173	80	503	573	312
By 3-month intervals :															
Inclusion	102	123	72	100	115	71	100	122	70	99	116	72	102	123	72
<=3 months	1	0	0	0	0	0	0	0	0	0	0	0	1	0	0
3-6 months	8	0	0	2	0	0	0	0	0	0	0	0	7	0	0
6-9 months	23	57	24	12	56	24	0	56	21	0	0	0	23	57	25
9-12 months	28	38	17	10	38	17	0	38	17	0	0	0	30	36	16
12-15 months	39	36	23	18	33	23	0	35	20	0	0	0	41	37	24
15-18 months	41	62	25	24	61	23	0	59	20	0	0	0	44	58	29
18-21 months	30	26	24	17	27	25	4	26	21	0	0	0	31	26	26
21-24 months	42	44	22	28	45	22	14	45	14	0	0	0	42	47	22
24-27 months	42	38	11	33	38	12	22	37	10	1	0	1	42	40	15
27-30 months	33	37	13	18	37	13	6	35	9	0	2	0	32	40	11
30-33 months	28	27	6	16	26	6	11	25	5	5	9	2	28	28	7
33-36 months	33	25	13	32	25	11	28	25	7	23	17	1	34	25	13
36-39 months	0	8	5	0	9	5	0	9	3	0	9	1	0	9	5
39-42 months	1	2	4	1	2	4	1	2	2	0	2	0	1	2	5
42-45 months	1	1	8	1	1	7	1	1	6	0	0	0	1	1	9
45-48 months	4	2	2	4	2	2	4	1	0	3	0	0	4	2	3
48-51 months	17	14	10	17	14	9	17	13	1	12	8	0	18	14	10
51-54 months	15	20	8	15	20	6	15	15	7	10	8	3	16	20	12
54-57 months	6	4	8	6	4	8	6	4	6	4	2	0	6	8	8

Web Figure 1. Flowchart of the subjects included the analysis from the initial COGICARE study

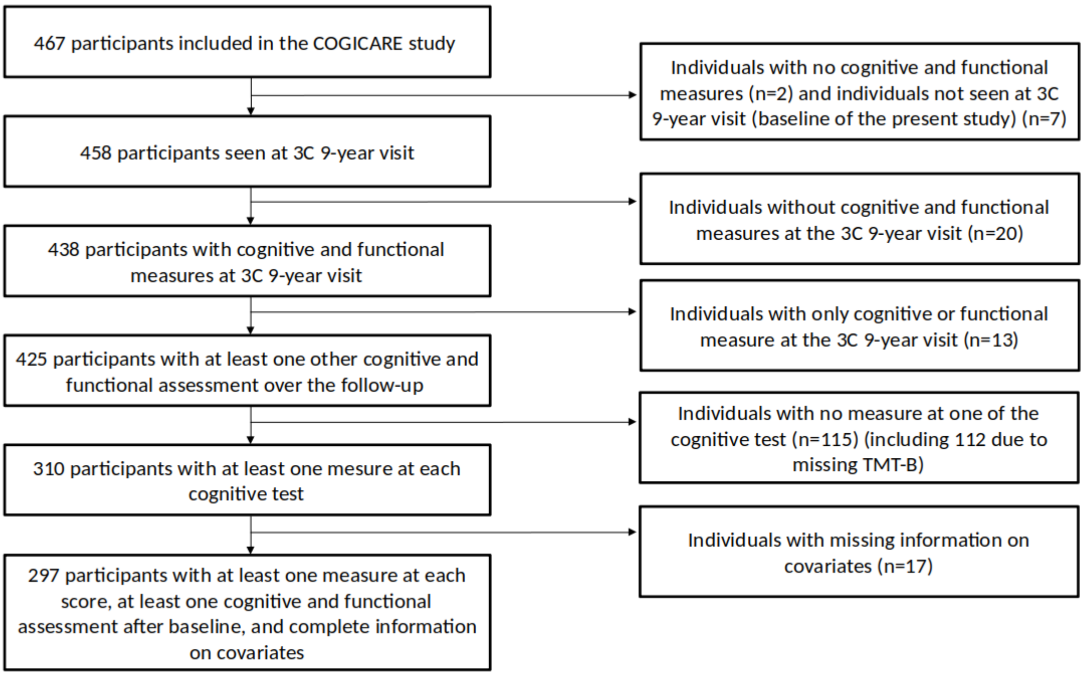

Web Figure 2: Individual trajectories and averaged trajectory of cognitive and functional measures from the Cogicare study (n=297), France

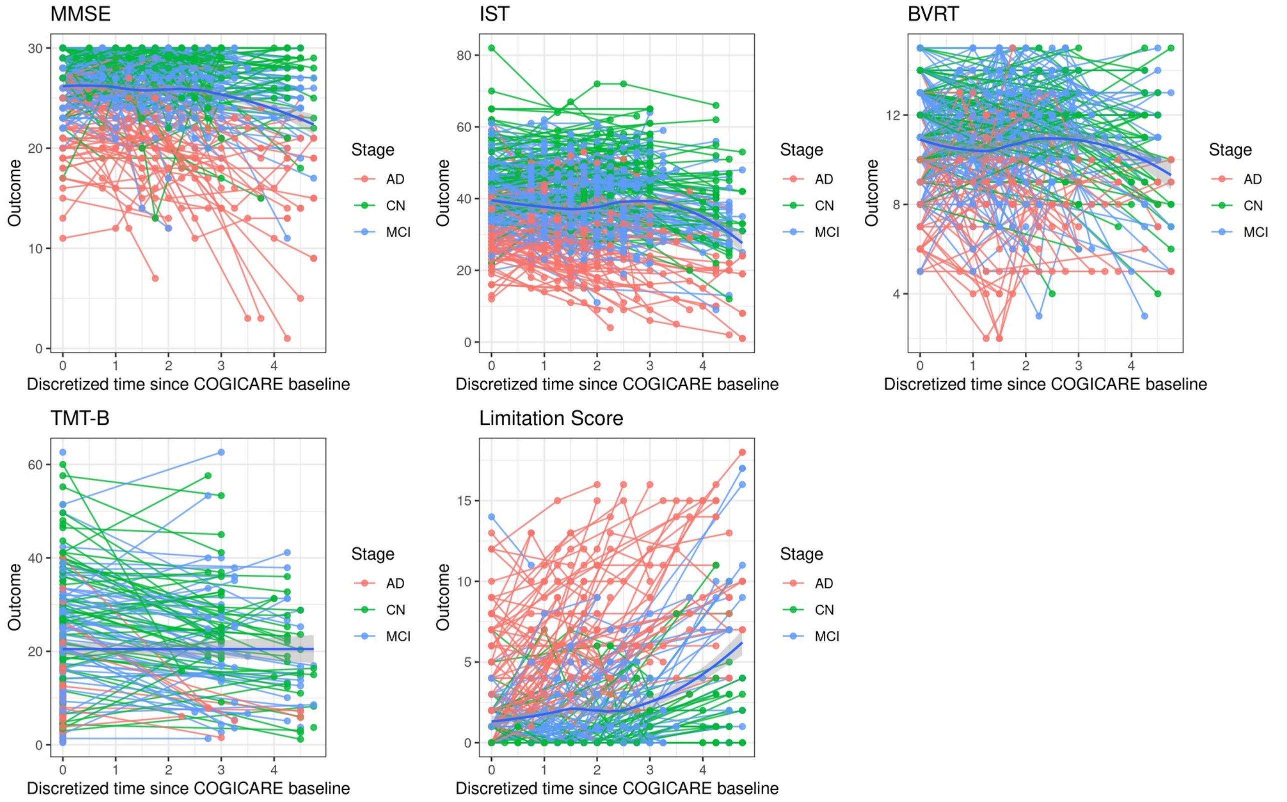

Web Figure 3: Estimated link functions between the latent cognitive dimension and the observed neuropsychological scores : MMSE, IST, BVRT, TMT-B(x10) (on the left) and between the latent functional level and the observed limitation score at IADL and BADL. 95% confidence intervals were obtained using a Monte Carlo method with 2000 draws.

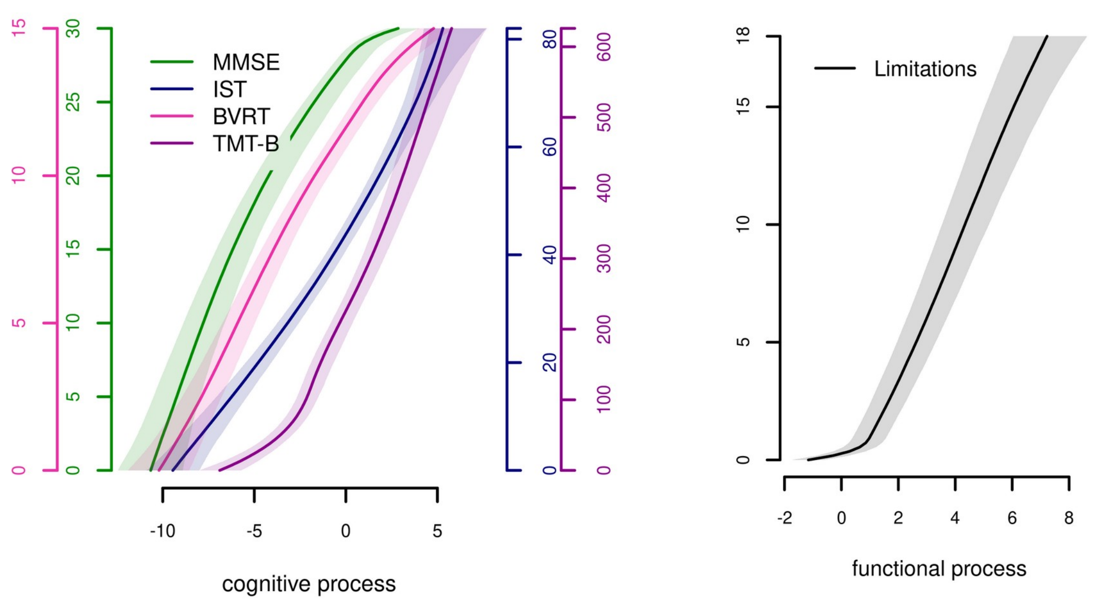

Web Figure 4: Predicted (plain blue line) and Observed (black dots) transformed marker values averaged by 6-month intervals in each clinical stage (CN, MCI, AD). Transformed observations correspond to the observations transformed by the estimated link function.

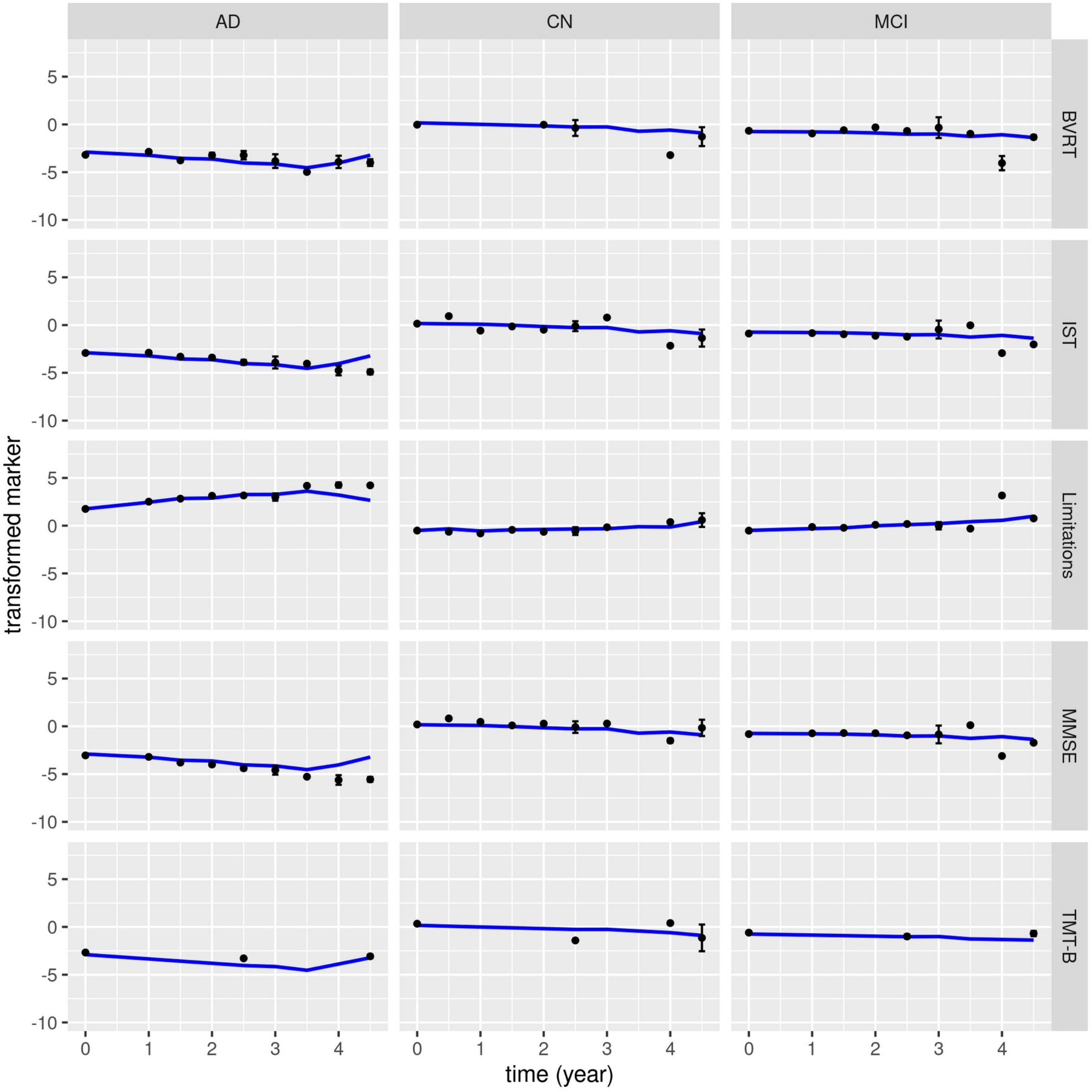

Supplement: Supplementary file 1 — Additional file 1. [file 13195_2021_887_MOESM1_ESM.pdf]
